# Birth order and parental and sibling involvement in sex education. A nationally-representative analysis

**DOI:** 10.1080/14681811.2018.1509305

**Published:** 2018-09-27

**Authors:** Lotte Elton, Melissa Palmer, Wendy Macdowall

**Affiliations:** a Department of Population Health, London School of Hygiene and Tropical Medicine, London, UK; b Department of Public Health, Environments and Society, London School of Hygiene and Tropical Medicine, London, UK

**Keywords:** Birth order, sex education, family, siblings, sexual health, Britain

## Abstract

This analysis set out to identify associations between birth order and sexual health outcomes, focusing on family involvement in sex education and early sexual experiences. The third National Survey of Sexual Attitudes and Lifestyles is a stratified probability sample survey of 15 162 men and women aged 16–74 in Britain. Logistic regression was conducted to identify odds ratios for the association between birth order and sexual health outcomes. Multiple logistic regression was performed adjusting for socio-demographic factors and sibling number. Middle-born and last-born men had lower odds of reporting ease talking to parents about sex around age 14 and learning about sex from their mothers. Last-born women had lower odds of reporting a parental main source of sex education or having learned about sex from their mother. Findings represent an exploratory analysis in an under-researched area, and provide the basis for further research on the association between birth order and parental involvement in sex education, as well as the role and impact of sex education provided by older siblings.

## Background

The construct of birth order has been extensively discussed over the last century in both public and academic spheres, with the aim of dissecting how the order in which children are born into a family may impact psychological or social outcomes. Theories of birth order stress the differing psychosocial environment experienced by each ordinal birth position, emphasising the quest of children to court the attention and approval of their parents and find a ‘niche’ amongst their siblings (Adler 1928; Sulloway 1996). Such processes are thought to account for the differences by birth order in measures such as achievement (Booth and Kee 2009) and conformity (Becker and Carroll 1962; Bragg and Allen 1970).

In spite of the proliferation of birth order literature, studies assessing the impact of birth order on sexual health outcomes are limited in number, and have mostly focussed on one outcome such as age at first sex (Miller et al. 1987; Rodgers, Rowe, and Harris 1992) or on sexual health in the context of risk-taking behaviour (Argys, Rees, Averett, and Witoonchart, 2010). Although research into aspects of birth order as they relate to sexual health is lacking, by extrapolating from existing birth order theories it is possible to hypothesise that there are a number of sexual health outcomes which birth order may influence. This analysis utilises a large probability sample survey of British men and women to investigate the associations between birth order and two aspects of sexual health: family involvement in sex education and early sexual experiences.

Numerous studies have highlighted the effect of birth order on parental treatment of children: Ng, Mofrad, and Uba (2014), for example, found significant differences in parental control between first- and last-born children. First-born children are described as being under increased pressure to conform to adults’ expectations (Baskett 1985) and are more likely to report higher levels of parental control and influence, with parental discipline weakening from first-born to last-born (Hotz and Patano 2015). First-borns also appear to have stronger familial sentiments: Salmon and Daly (1998) identify first-borns as more likely than middle- or last-borns to nominate parents as figures they would turn to under duress, and to choose their mothers as the person to whom they feel closest. Middle-borns, in particular, appear to have markedly less close relationships with their parents, with Kidwell (1981) finding that middle-borns were significantly less likely than first- or last-borns to report parental reasonableness and supportiveness.

One aspect of the parent-child relationship concerns the involvement of parents in educating their children about sexual matters. Parental involvement in sex education appears to have a protective effect on their child's sexual health: reporting a parent as a main source of sex education has been associated with decreased odds of a number of sexual risk behaviours, including having had unsafe safe in the past year (defined as no condom use on first occasion with a new partner) (Macdowall et al. 2015) and having low self-efficacy for condom negotiation (Crosby, Hanson, and Rager 2009). Other facets of parental interaction also seem to bear a relationship to the sexual behaviour of children, with increased parental monitoring (Hogan and Kitagawa 1985; Rodgers, Rowe, and Harris 1992; Small and Luster 1994) and parental support (Chewning and Koningsfeld 1998; Feldman and Brown 1993) both being associated with later age at first intercourse.

The ease and frequency of communication between parents and children about sexual matters has been shown to vary according to a number of factors, with openness of the parent-child relationship (Wilson et al. 2010) and self-esteem of children (Salazar et al. 2005) both positively correlated with parental involvement in sex education. Several studies have reported a strong association between gender and parental sex education, with three times as many women as men reporting a parent as their main source of sex education (Macdowall et al. 2006; Tanton et al. 2015). This is corroborated by qualitative research which suggests that mothers – who are far more likely to be the main sex educators in a household – feel more comfortable discussing sexual matters with their daughters (Walker 2001).

Little research has so far linked birth order and family involvement in sex education. What evidence there is for birth order effects on sex education derives mostly from qualitative research: Turnbull (2012), for example, noted the presence of younger siblings as constituting a barrier to open communication about sexual matters. However, Turnbull also found sexual matters were more likely to be discussed openly when parents and children spend more time together, and that parents resumed conversations about sex with their first-born children at a time when they could be alone; extrapolating from previous birth order research which suggests that first-born and only children receive a greater degree of parental attention (Collins 2006) it could be hypothesised that parents are more likely to discuss sexual matters with their first-born children.

### Rationale for analysis

Despite a wide range of literature addressing parental involvement in sex education, little research has been undertaken to assess how birth order affects communication about sex with parents; this seems a salient area for investigation given the well-documented effects of the parent-child relationship on learning about sex from one’s parents.

Much of the literature around birth order and sexual risk-taking behaviour focuses on risk-taking during adolescence, at a time when first sexual experiences are most likely to occur (Wellings et al. 2001). Early sexual experiences appear to be important in determining later sexual health outcomes: previous research has established a correlation between competence at first heterosexual intercourse – defined as use of contraception, autonomy of decision, equal willingness of both partners, and a belief that it happened at the ‘right time’ – and a variety of sexual health outcomes, including low sexual function, and, among women, ever having had an STI diagnosis (Palmer et al. 2016). Similarly, ‘early’ sex – occurring before the age of 16 – is a predictor of subsequent poor sexual health (Royal College of Physicians 2011). The present analysis therefore includes investigation of early sexual experiences for two reasons: first, because early sexual experiences commonly occur around late adolescence, when individuals are likely to be living in their family home and thus may be more susceptible to family influences; secondly, because early sexual experiences may correlate with or have a bearing upon later risk-taking behaviour.

In this study, we analysed data from the British National Survey of Sexual Attitudes and Lifestyles using univariable and multiple logistic regression, in order identify any association between birth order and sexual health outcomes. We set out to compare the characteristics of those who are first-born with those of middle-borns and last-borns in two key areas: familial involvement in sex education and early sexual experiences. Based on previous studies relating to birth order, parental involvement and risk-taking behaviour, we hypothesised that first-born children would be more likely to report parents as a source of sex education and less likely to report negative outcomes at first sexual intercourse. Given the dearth of literature on birth order and sexual health, this was an exploratory analysis, and aimed to identify salient areas for further study. In assessing whether birth order has any impact on sexual health outcomes, we aimed to lay the path for further research into how key theories of birth order differences might be applied in the field of sexual health, as well as to consider how public health measures might be tailored to address birth-order related disparities in sexual health outcomes.

## Method

### Study design

The third National Survey of Sexual Attitudes and Lifestyles (Natsal-3) is a multi-stage, clustered and stratified probability sample survey of 15 162 men and women aged 16–74 resident in Britain, and was conducted between the September 2010 and August 2012. Natsal-3 is the most recent iteration of a decennial national probability sample of sexual behaviour in the Britain; along with its earlier counterparts Natsal-1 and Natsal-2, it is amongst the largest and most thorough sexual behaviour surveys worldwide. Full details of the methodology have been published elsewhere in Erens et al. (2013).

### Sampling and questionnaire

The Natsal-3 study aimed to conduct 15 000 interviews in total, of which 10 000 would be a ‘core’ sample (adults aged 16–74) and 5 000 a ‘boost’ sample (adults aged 16–34). Participants in this younger age group were oversampled using two boost samples (boost 1, between ages 16–34, and boost 2, between ages 16–29) to give sufficient statistical power to explore in detail the sexual behaviours of young people, who are at the highest risk of a variety several sexual health outcomes such as STIs. The response rate was 57.7% for the whole sample, 64.8% for boost 1 and 67.3% for boost 2 (Erens et al. 2014). As in Natsal-2, Natsal-3 used a combination of face-to-face interview (using computer-assisted personal interviews (CAPI)) and self-completion, using computer-assisted self-interviews (CASI). More sensitive questions – such as those covering experiences of different sexual practices – were asked in the CASI (Mercer et al. 2013).

### Demographic and explanatory variables

For our analysis, the explanatory variable of interest was birth order, which was coded as ‘first-born’, ‘middle-born’ and ‘last-born’. This variable was derived from a question which asked ‘Thinking about the siblings you lived with when you were growing up, were you the…. Oldest, youngest or in-between?’ 10.7% of the our analysis sample had a response of ‘not applicable’ to this question, either because they were an only child or because they did not live with their siblings at any time when growing up. Since it was not possible to determine from the data the birth order of those who did not live with any of their siblings at any time when growing up, these participants (4.2% of the analysis sample), along with only children (6.5% of the analysis sample) were excluded from the analyses.

### Outcome variables

Based on a literature review, outcome variables relating to familial involvement in sex education and early sexual experiences were selected from the Natsal-3 data. The variable denoting the ease with which respondents spoke about sex with their parents was recoded to give a binary variable, with *Easy with one/both* recoded as ‘easy to discuss sex with parents’ and *Difficult with one/both, Didn’t discuss with either*, and *Varied/depended on the topic* recoded as ‘not easy to discuss sex with parents’. The continuous variable for age at first heterosexual intercourse was recoded to give a binary variable for ‘early’ sex (<16 and ≥16) (Wellings et al. 2001). A variable for age difference between partners at first sex was newly generated from variables for participant age at first sex and the age of their partner at first sex, and then recoded into binary variables for ‘partner relatively younger at first sex’ and ‘partner relatively older at first sex’. As in Mercer et al. (2006), ‘relatively younger’ and ‘relatively older’ were defined separately for men and women using the cohort-specific 5^th^ and 95^th^ percentiles of age difference at first sex. Relatively older partners were defined as 3 years older for men and 7 years older for women; relatively younger partners were defined as 2 years younger for men and 1 year younger for women.

Other categorical variables were recoded into binary variables for the outcome of interest – for example, a new binary variable for autonomous reasoning for first heterosexual intercourse was generated, which designated ‘peers doing it’ or being under the influence of alcohol/drugs as non-autonomous reasons. A variable for ‘sexual competence’ was generated from four variables relating to circumstances of first sex: use of contraception (defined as use of a condom, hormonal contraception, or non-hormonal contraceptive device), willingness, perceived timing and autonomy, such that ‘competence’ implies use of contraception, both participants equally willing, perceived right timing, and an autonomous reason for first sex. In all cases, the recoding of categorical variables was conducted so as to remain faithful to the original answer, with the aim of increasing analytical power and measurement validity.

### Statistical analyses

Statistical analyses were conducted using STATA 14.1, using survey functions in order to account for the weighting, clustering, and stratification in the Natsal-3 dataset. Since many of the outcomes of interest related to events that may have been some time ago, such as first sexual experiences, analysis was restricted to participants aged 17–29 at interview in order to reduce recall bias. The use of a younger sample also allowed for a clearer determination of associations between birth order and risk behaviours which were less likely to be confounded by later life events. Those aged 16 (n = 446) were excluded, as in Macdowall et al. (2015), since these participants may not have yet finished their compulsory education and could not be ascribed an educational level, a variable which was included in the multiple logistic regression as a potential confounder. The proportion of non-response values for each variable was investigated; since non-response was low for all variables of interest, participants with missing data were omitted from analyses.

Socio-demographic characteristics by birth order and sex were analysed and are shown in Tables 1 and 2. Those characteristics which were associated with any birth order category with p < 0.05 were considered potential confounders. These characteristics were education level, ethnic group, religion, quintile of adjusted Index of Multiple Deprivation (IMD), parents’ social class, and family structure at age 14. The variable for parents’ social class was based on participant reported parental occupations (as outlined in Table 1). The IMD is a measure of relative deprivation for small areas or neighbourhoods, taking into account factors such as income, employment, education, and living environment (Payne and Abel 2012).

**Table 1.** Socio-demographic characteristics by birth order in women aged 17–29 in Natsal-3.

	Women			
Characteristics	First-born	Middle-born	Last-born	p
Birth order by % of respondents	37.9% (35.9 – 39.8)	26.2% (24.5 – 28.1)	35.9% (33.9 – 37.9)	
Denominator (weighted, unweighted [W, UW])	611, 1124	424, 763	580, 1075	
**Age at interview**				0.0097
17–19	23.1% (20.6 – 25.9)	18.3% (15.4 – 21.7)	22.4% (19.8 – 25.3)	
20–24	40.6% (37.2 – 44.0)	40.8% (36.9 – 44.8)	35.4% (32.2 – 38.7)	
25–29	36.3% (33.1 – 39.6)	40.9% (37.1 – 44.7)	42.2% (38.9 – 45.5)	
Denominator (W, UW)	611, 1124	424, 763	580, 1075	
**Highest academic qualification**				< 0.0001
No academic qualifications	5.3% (4.1 – 6.8)	10.3% (8.2 – 12.7)	5.8% (4.5 – 7.4)	
Qualifications expected at age 16	24.9% (22.2 – 27.8)	32.6% (29.1 – 36.4)	27.7% (24.8 – 30.8)	
Studying for further education	69.8% (66.7 – 72.7)	57.1% (53.1 – 61.0)	66.5% (63.2 – 69.7)	
Denominator (W, UW)	591, 1088	410, 739	553, 1035	
**Ethnicity**				< 0.0001
White	82.8% (79.8 – 85.4)	73.6% (69.3 – 77.5)	88.2% (85.6 – 90.3)	
Mixed	4.5% (3.2 – 6.3)	4.1% (2.8 – 5.8)	3.0% (2.0 – 4.4)	
Asian/Asian British	8.2% (6.2 – 10.7)	11.9% (9.0 – 15.5)	5.6% (4.2 – 7.4)	
Black/Black British	3.6% (2.5 – 5.2)	8.1% (6.0 – 10.8)	2.6% (1.6 – 4.2)	
Chinese/Other	0.9% (0.4 – 1.9)	2.4% (1.3 – 4.3)	0.7% (0.3 – 1.3)	
Denominator (W, UW)	610, 1122	424, 763	579, 1073	
**Religion**				< 0.0001
None	58.1% (54.5 – 61.6)	52.2% (48.0 – 56.4)	59.8% (56.6 – 63.1)	
Christian (all)	32.3 (29.2 – 35.6)	32.9% (29.2 – 36.8)	34.0% (30.7 – 37.3)	
Muslim	5.2% (3.6 – 7.4)	11.6% (8.8 – 15.3)	3.3% (2.3 – 4.8)	
Hindu	2.3% (1.5 – 3.7)	1.2% (0.6 – 2.3)	1.0% (0.6 – 1.8)	
Non-Christian other	2.1% (1.2 – 3.4)	2.1% (1.1 – 3.9)	1.9% (1.2 – 3.1)	
Denominator (W, UW)	610, 1122	423, 762	578, 1072	
**Parents’ social class**^1^				< 0.0001
I/II/III	71.8% (68.7 – 74.8)	63.7% (59.1–67.5)	73.5% (70.4 – 76.4)	
IV/V	19.1% (16.5 – 22.0)	22.0% (18.9 – 25.4)	18.4% (15.9 – 21.1)	
Not answered*	9.1% (7.5 – 11.0)	14.4% (11.8 – 17.4)	8.2% (6.5 – 10.2)	
Denominator (W, UW)	598, 1092	411, 734	570, 1052	
**Quintile of adjusted index of multiple deprivation**				< 0.0001
1 (least deprived)	15.9% (13.4 – 18.8)	11.2% (9.0 – 13.8)	17.6% (15.0 – 20.6)	
2	16.8% (14.2 – 19.7)	12.9% (10.3 – 16.0)	18.4% (15.9 – 21.2)	
3	21.2% (18.5 – 24.2)	18.6% (15.5 – 22.2)	20.4% (17.5 – 23.7)	
4	23.4% (20.6 – 26.4)	24.0% (20.6 – 27.9)	22.2% (19.2 – 25.5)	
5 (most deprived)	22.7% (20.0 – 25.7)	33.3% (29.3 – 37.6)	21.4% (18.6 – 24.5)	
Denominator (W, UW)	611, 1124	424, 763	580, 1075	
**Family structure at age 14**				0.0007
Both natural parents	68.8% (65.7 – 71.7)	66.1% (62.2 – 69.7)	74.7% (71.8 – 77.4)	
One natural parent	29.5% (26.6 – 32.5)	31.3% (27.8 – 33.0)	24.4% (21.7 – 27.2)	
Neither natural parent	1.8% (1.2 – 2.6)	2.6% (1.7 – 4.1)	0.9% (0.5 – 1.6)	
Denominator (W, UW)	611, 1124	423, 762	580, 1075	
**Number of siblings**				< 0.0001
1	44.5% (41.2 – 47.9)	0	43.7% (40.5 – 47.0)	
2	23.7% (20.9 – 26.7)	29.2% (25.7 – 33.0)	25.9% (23.0 – 29.0)	
3	14.3% (12.0 – 17.0)	26.7% (23.4 – 30.3)	13.9% (11.8 – 16.2)	
4+	17.5% (15.3 – 20.0)	44.1% (40.2 – 48.0)	16.6% (14.3 – 19.2)	
Denominator (W, UW)	611, 1124	424, 762	580, 1074	

* Included here because the non-response rate for this question was high (9.1%) and varied by birth order status. All other variables had non-response rates < 5% (usually 0.5–2%) which were not differential according to birth order.

^1^Parents’ social class was based on participant-reported parental occupation as follows: I – Professional; II – Managerial and technical; III – Skilled non-manual or manual; IV – Partly-skilled; and V – Unskilled.

**Table 2.** Socio-demographic characteristics by birth order in men aged 17–29 in Natsal-3.

	Men			
Characteristics	First-born	Middle-born	Last-born	p
Birth order by % of respondents	39.5% (37.3 – 41.8)	24.4% (22.3 – 26.5)	36.1% (33.9 – 38.4)	
Denominator (weighted, unweighted [W, UW])	648, 849	399, 485	592, 801	
**Age at interview**				0.9844
17–19	22.4% (19.5 – 25.7)	21.6% (17.9 – 25.9)	23.1% (20.1 – 26.3)	
20–24	37.7% (34.1 – 41.5)	39.2% (34.5 – 44.2)	38.8% (35.0 – 42.8)	
25–29	39.8% (36.2 – 43.6)	39.1% (34.3 – 44.2)	38.1% (34.3 – 42.1)	
Denominator (W, UW)	648, 849	399, 485	592, 801	
**Highest academic qualification**				< 0.0001
No academic qualifications	5.2% (3.8 – 7.1)	11.9% (8.9 – 15.7)	6.1% (4.5 – 8.2)	
Qualifications expected at age 16	27.3% (24.0 – 30.8)	35.4% (30.8 – 40.4)	26.7% (23.4 – 30.2)	
Studying for further education	67.5% (63.8 – 70.8)	52.7% (47.5 – 57.9)	67.3% (63.5 – 70.9)	
Denominator (W, UW)	626, 825	382, 469	568, 776	
**Ethnicity**				0.0046
White	85.2% (81.9 – 88.0)	76.7% (71.8 – 80.9)	84.9% (81.6 – 87.7)	
Mixed	2.3% (1.3 – 4.0)	4.1% (2.6 – 6.5)	2.8% (1.7 – 4.6)	
Asian/Asian British	7.2% (5.3 – 9.7)	11.9% (8.5 – 16.4)	9.0% (6.8 – 11.8)	
Black/Black British	3.0% (2.0 – 4.4)	5.6% (3.5 – 8.7)	2.2% (1.4 – 3.5)	
Chinese/Other	2.4% (1.4 3.8)	1.7% (0.8 – 3.5)	1.1% (0.6 – 2.0)	
Denominator (W, UW)	648, 849	399, 485	591, 799	
**Religion**				0.0005
None	63.1% (59.2 – 66.8)	57.5% (52.4 – 62.5)	64.1% (60.0 – 68.0)	
Christian (all)	28.1% (24.9 – 31.5)	27.8% (23.4 – 32.5)	26.6% (23.1 – 30.4)	
Muslim	4.8% (3.3 – 6.9)	11.1% (7.9 – 15.5)	5.1% (3.4 – 7.5)	
Hindu	2.1% (1.1 – 4.01)	0.5% (0.1 – 2.0)	2.8% (1.7 – 4.5)	
Non-Christian other	2.0% (1.1 – 3.4)	3.1% (1.8 – 5.5)	1.4% (0.8 – 2.5)	
Denominator (W, UW)	647, 848	399, 485	591, 798	
**Parents’ social class**				0.0074
I/II/III	76.3% (73.2 – 79.3)	69.0% (63.9 – 73.6)	73.7% (70.2 – 76.9)	
IV/V	14.7% (12.3 – 17.5)	18.4% (14.7 – 22.7)	19.1% (16.2 – 22.3)	
Not answered*	8.9% (7.1 – 11.2)	12.7% (9.7 – 16.4)	7.3% (5.6 – 9.4)	
Denominator (W, UW)	636, 831	388, 469	585, 789	
**Quintile of adjusted index of multiple deprivation**				0.0009
1 (least deprived)	16.6% (13.8 – 19.9)	13.1% (10.0 – 16.9)	17.9% (15.1 – 21.0)	
2	19.6% (16.6 – 23.0)	15.6% (12.2 – 19.7)	19.7% (16.6 – 23.3)	
3	16.3% (13.6 – 19.3)	15.6% (12.4 – 19.5)	21.7% (18.5 – 25.3)	
4	24.5% (21.0 – 28.4)	26.6% (21.9 – 32.0)	19.6% (16.2 – 23.5)	
5 (most deprived)	23.0% (19.8 – 26.7)	29.1% (24.6 – 34.2)	21.1% (18.0 – 24.7)	
Denominator (W, UW)	648, 849	399, 485	592, 801	
**Family structure at age 14**				0.0332
Both natural parents	74.1% (70.7 – 77.2)	73.3% (68.8 – 77.3)	78.9% (75.6 – 81.8)	
One natural parent	24.8% (21.8 – 28.1)	24.5% (20.6 – 28.9)	20.4% (17.5 – 23.7)	
Neither natural parent	1.1% (0.6 – 2.1)	2.2% (1.2 – 4.0)	0.7% (0.3 – 1.6)	
Denominator (W, UW)	648, 849	399, 485	592, 801	
**Number of siblings**				< 0.0001
1	46.3% (42.4 – 50.3)	0	46.9% (43.5 – 50.8)	
2	27.6% (24.3 – 31.1)	36.8% (32.1 – 41.7)	27.3% (23.9 – 31.0)	
3	14.3% (11.9 – 17.0)	28.1% (23.8 – 32.9)	12.6% (10.1 – 15.5)	
4+	11.9% (9.8 – 14.5)	35.1% (30.5 – 40.0)	13.3% (10.8 – 16.3)	
Denominator (W, UW)	647, 848	399, 485	592, 801	

* Included here because the non-response rate for this question was high (9.1%) and varied by birth order status. All other variables had non-response rates < 5% (usually 0.5–2%) which were not differential according to birth order.

Odds ratios were calculated which were first adjusted for socio-demographic confounding variables, except for outcomes based on questions about learning about sex which specified ‘at age 14’: here, education level was removed from the multiple logistic regression model, since it was unlikely to be a confounding factor. A further key confounder was family size: since all middle-born children must, by definition, come from a family with at least 3 children, they were therefore more likely to come from larger families, on average, than the first- or last-borns in the study. The multiple regression model was thus extended to include sibling number as a measure of family size, and fully-adjusted odds ratios were calculated.

Sibling number was defined as the number of siblings an individual participant co-resided with for at least part of their life; a variable for 1, 2, 3 or 4+ siblings was used. A significance level of 0.05 was used in the multiple logistic regression model to determine whether birth order was independently associated with an outcome.

### Ethical approval

This project was approved by the London School of Hygiene and Tropical Medicine’s combined academic, risk assessment and ethics (CARE) committee. Ethical approval for Natsal-3 was granted by the Oxfordshire Research Ethics Committee A (reference number 10/H0604/27) (Erens et al. 2013).

## Results

The unweighted subsample consisted of 2135 men and 2962 women aged between 17 and 29. The proportions of first-born, middle-born, and last-born children did not differ significantly between the sexes (p = 0.3314). Amongst women, 37.9% were first-born, 26.2% middle-born, and 35.9% last-born. Amongst men, 39.5% were first-born, 24.4% middle-born, and 36.1% last-born.

### Socio-demographic factors

There were associations between birth order and educational level, ethnicity, religion, parents' social class, quintile of adjusted index of multiple deprivation, and family structure at age 14, and number of siblings (all p < 0.05) (Tables 1 and 2). Compared to first- or last-born women, a higher proportion of middle-born women had no academic qualifications, were of Black/Black British ethnicity, were Muslim, and were living in an area in the lowest quintile of adjusted index of multiple deprivation. Compared to first- or last-born men, a higher proportion of middle-born men had no academic qualifications and were Muslim. In both men and women, a greater proportion of middle-borns had 2, 3, or 4+ siblings when compared to first- or last-borns.

### Learning about sex

First-borns, whether male or female, reported parental involvement in sex education in the highest proportions (Table 3). 48.0% of first-born women and 37.3% of first-born men reported discussing sex with 1+ parent around age 14, compared to 39.9% of middle-born women and 29.0% of last-born men. Amongst men, a smaller proportion of middle-borns reported ease talking to parents about sex when compared with first-borns. Amongst men and women, a smaller proportion of middle-borns reported learning about sex from their mother when compared with first-borns. Amongst men, a smaller proportion of middle-borns reported ease talking to parents about sex when compared with first-borns. Amongst men and women, a smaller proportion of middle-borns reported learning about sex from their mother when compared with first-borns.

**Table 3.** Learning about sex by birth order, for men and women aged 17–29 in Natsal-3.

Outcome variable	Women		Men	
	% (95% CI)	Denominators (W, UW)	% (95% CI)	Denominators (W, UW)
**Found it easy to talk to parents about sex around age 14**				
First-born	32.6% (29.6 – 35.7)	599, 1092	27.9% (24.5 – 31.6)	640, 835
Middle-born	29.4% (25.7 – 33.3)	411, 738	17.8% (14.5 – 21.8)	390, 472
Last-born	30.9% (27.8 – 34.2)	574, 1059	21.4% (18.0 – 25.1)	585, 790
**Discussed sex with 1+ parent(s) around age 14**				
First-born	48.0% (44.7 – 51.4)	599, 1092	37.3% (33.6 – 41.2)	640, 835
Middle-born	39.9% (36.1 – 43.8)	411, 738	29.0% (24.6 – 33.8)	390, 472
Last-born	45.0% (41.7 – 48.5)	574, 1059	34.1% (30.4 – 38.1	585, 790
**Learned about sex from mother**				
First-born	45.8% (42.5 – 49.2)	611, 1124	24.5% (21.4 – 27.9)	648, 849
Middle-born	35.9% (32.3 – 39.8)	424, 763	14.0% (11.0 – 17.7)	399, 484
Last-born	41.7% (38.4 – 45.0)	579, 1074	20.7% (17.5 – 24.3)	592, 801
**Learned about sex from father**				
First-born	7.4% (5.8 – 9.3)	611, 1124	19.6% (16.8 – 22.8)	648, 849
Middle-born	6.9% (5.1 – 9.3)	424, 763	17.3% (13.9 – 21.5)	399, 484
Last-born	6.8% (5.2 – 8.7)	579, 1074	15.7% (13.0 – 19.0)	592, 801
**Learned about sex from siblings**				
First-born	1.1% (0.6 – 2.0)	611, 1124	1.4% (0.7 – 3.0)	648, 849
Middle-born	18.0% (15.0 – 21.3)	424, 763	16.4% (13.2 – 20.2)	399, 484
Last-born	19.3% (16.7 – 22.1)	579, 1074	18.5% (15.5 – 21.9)	592, 801
**Main source of sex education was a parent**				
First-born	16.9% (14.6 – 19.4)	608, 1119	8.6% (6.6 – 11.0)	647, 846
Middle-born	13.7% (11.3 – 16.6)	421, 757	6.8% (4.7 – 9.7)	398, 483
Last-born	11.6% (9.7 – 13.9)	576, 1067	6.1% (4.3 – 8.6)	588, 794

Table 4 shows the results of multiple logistic regression analysis: partially-adjusted odds ratios, adjusted for socio-demographic factors, are shown alongside fully-adjusted odds ratios which include adjustment for sibling number. Both middle-born and last-born men had reduced odds of reporting having found it easy to speak to their parents about sex around age 14 (OR 0.58, 95% CI 0.41 – 0.83; OR 0.69, 95% CI 0.52 – 0.91, respectively) and to have learned about sex from their mothers (OR 0.62, 95% CI 0.42 – 0.89; OR 0.75, 95% CI 0.57 – 0.99, respectively). Middle-born men were also less likely to have discussed sex with 1 or more parent around age 14 (OR 0.72, p = 0.032). In women, being last-born was associated with reduced odds of learning about sex from one’s mother (OR 0.80, 95% CI 0.65 – 0.97) and reporting at least one parent as a main source of sex education (OR 0.62, 95% CI 0.47 – 0.83).

**Table 4.** Learning about sex by birth order in women and men aged 17–29 in Natsal-3 – multiple regression models adjusting for socio-demographic factors and family size.

	Women				Men			
Outcome variable	paOR* (95% CI)	P	faOR** (95% CI)	P	paOR* (95% CI)	P	faOR** (95% CI)	P
**Found it easy to talk to parents about sex around age 14**								
First-born	1.00	0.6078	1.00	0.5810	1.00	0.0048	1.00	0.0032
Middle-born	0.95 (0.75 – 1.21)		0.99 (0.76 – 1.29)		0.62 (0.45 – 0.86)		0.58 (0.41–0.83)	
Last-born	0.90 (0.73 – 1.10)		0.90 (0.72 – 1.11)		0.69 (0.52 – 0.92)		0.69 (0.52–0.92)	
**Discussed sex with 1+ parent(s) around age 14**								
First-born	1.00	0.0630	1.00	0.0974	1.00	0.1899	1.00	0.0931
Middle-born	0.79 (0.62 – 0.99)		0.82 (0.64 –1.04)		0.77 (0.58 – 1.03)		0.71 (0.52–0.97)	
Last-born	0.82 (0.67 – 1.00)		0.82 (0.67 – 1.00)		0.87 (0.68 – 1.10)		0.87 (0.68 – 1.10)	
**Learned about sex from mother**								
First-born	1.00	0.0531	1.00	0.0723	1.00	0.0041	1.00	0.0163
Middle-born	0.82 (0.65 – 1.02)		0.84 (0.66 – 1.08)		0.59 (0.42 – 0.82)		0.62 (0.42 – 0.89)	
Last-born	0.79 (0.65 – 0.97)		0.80 (0.65 – 0.97)		0.75 (0.57 – 0.99)		0.75 (0.57 – 0.99)	
**Learned about sex from father**								
First-born	1.00	0.5222	1.00	0.5338	1.00	0.1309	1.00	0.0590
Middle-born	1.13 (0.74 – 1.72)		1.13 (0.72 – 1.77)		1.08 (0.76 – 1.53)		1.21 (0.82 – 1.80)	
Last-born	0.87 (0.60 – 1.27)		0.86 (0.59 – 1.25)		0.77 (0.56–1.05)		0.76 (0.56 – 1.04)	
**Learned about sex from siblings**								
First-born	1.00	<0.0001	1.00	<0.0001	1.00	<0.0001	1.00	<0.0001
Middle-born	18.45 (9.50 – 35.86)		14.36 (7.30 – 28.25)		15.83 (6.92 – 36.25)		14.20 (6.11 – 32.90)	
Last-born	21.45 (11.33 – 40.58)		21.67 (11.51 – 40.81)		17.53 (8.05 – 38.19)		17.60 (8.09 – 38.49)	
**Main source of sex education was a parent**								
First-born	1.00	0.0059	1.00	0.0060	1.00	0.0324	1.00	0.0256
Middle-born	0.88 (0.65 – 1.20)		0.85 (0.61 – 1.18)		0.99 (0.61 – 1.60)		1.07 (0.60 – 1.90)	
Last-born	0.63 (0.47 – 0.84)		0.62 (0.47 – 0.83)		0.57 (0.36 – 0.89)		0.57 (0.36 – 0.89)	

* Partially-adjusted odds ratios: adjusted for age, religion, ethnic group, quintile of adjusted index of multiple deprivation, parents’ social class, and family structure at age 14 ** Fully-adjusted odds ratios: adjusted for all socio-demographic factors and for number of siblings.

### Early sexual experiences

Table 5 displays early sexual experiences by birth order. A higher proportion of middle-born women reported pregnancy before the age of 18 (13.1%, compared to 8.5% of first-borns and last-borns).

**Table 5.** Early sexual experiences by birth order, for men and women aged 17–29 in Natsal-3.

Outcome variable	Women		Men	
	% (95% CI)	Denominators (W, UW)	% (95% CI)	Denominators (W, UW)
**Under 16 at first sex**				
First-born	25.9% (23 – 28.8)	597, 1102	24.7% (21.6 – 28.1)	638, 835
Middle-born	27.1% (23.9 – 30.6)	416, 750	37.7% (33.0 – 42.6)	395, 477
Last-born	29.6% (26.6 – 32.7)	568, 1051	25.0% (21.9 – 28.3)	582, 787
**Partner relatively older at first sex^a^**				
First-born	4.7% (3.4 – 6.2)	528, 990	7.4% (5.6 – 9.7)	553, 710
Middle-born	7.7% (5.9 – 10.1)	364, 680	5.7% (3.9 – 8.4)	351, 427
Last-born	6.3 (4.7 – 8.3)	508, 945	3.8% (2.5 – 5.7)	489, 669
**Either respondent or partner more willing at first sex^b^**				
First-born	19.2% (16.4 – 22.4)	529, 991	10.4% (7.8 – 13.7)	556, 715
Middle-born	19.5% (16.3 – 23.0)	366, 686	10.2% (7.5 – 13.8)	352, 429
Last-born	15.7% (13.3 – 18.5)	510, 951	7.3% (5.5 – 9.7)	491, 672
**Did not use reliable contraception at first sex**				
First-born	12.4% (10.3 – 14.8)	520, 970	13.4% (10.8 – 16.4)	555, 714
Middle-born	17.1% (14.2 – 20.3)	361, 673	19.7% (16.1 – 24.0)	350, 427
Last-born	9.6% (7.9 – 11.7)	507, 945	13.1% (10.5 – 16.4)	490, 670
**Thinks should have waited longer for first sex**				
First-born	33.0% (29.8 – 36.4)	518, 968	17.6% (14.7 – 20.9)	550, 709
Middle-born	39.0% (35.1 – 43.0)	358, 669	25.1% (20.7 – 30.1)	349, 424
Last-born	33.5% (30.3 – 36.8)	507, 945	16.0% (13.3 – 19.1)	490, 670
**Non-autonomous reason for first sex^c^**				
First-born	16.3% (13.9 – 18.9)	481, 907	12.7% (10.1 – 15.8)	529, 678
Middle-born	18.7% (15.5 – 22.4)	319, 602	14.2% (11.0 – 18.1)	334, 409
Last-born	15.3% (12.8 – 18.1)	478, 894	12.0% (9.6 – 15.0)	465, 642
**Not competent at first sex^d^**				
First-born	50.1% (46.6 – 53.6)	529, 990	42.1% (38.1 – 46.3)	551, 710
Middle-born	54.1% (49.8 – 58.4)	366, 685	52.6% (47.4 – 57.7)	350, 426
Last-born	47.7% (44.3 – 51.2)	510, 950	39.8% (35.7 – 44.0)	491, 672
**Pregnancy before age 18**				
First-born	8.5% (7.0 – 10.4)	565, 1018		
Middle-born	13.1% (10.8 – 15.8)	391, 705		
Last-born	8.5% (6.9 – 10.4)	523, 973		

^a^Relatively older partner defined as 3 years older for men and 7 years older for women.

^b^Excluding those who reported that their first experience of sexual intercourse was forced.

^c^Non-autonomous reasons for first sex were ‘peers doing it’ and being under the influence of alcohol or drugs.

^d^Sexual competence combines four variables relating to circumstances of first sex: use of contraception, willingness, perceived timing and autonomy, such that ‘competence’ implies use of contraception, both participants equally willing, perceived right timing, and an autonomous reason for first sex.

A higher proportion of middle-born men were non-competent at first sex (52.6%, compared to 42.1% of first-borns and 39.8% of last-borns) and had had first sexual intercourse under age 16 (37.7%, compared to 24.7% of first-borns and 25.0% of last-borns). Multiple regression adjusting for socio-demographic characteristics and sibling number demonstrated no statistically significant associations between birth order and early sexual experiences in women (Table 6). Being a middle-born male was associated with increased odds of being under 16 at first sexual intercourse (OR 1.82, 95% CI 1.31 – 2.52) while last-born men had reduced odds when compared to first-born men of reporting a relatively older partner at first sex (OR 0.75, 95% CI 0.38 – 1.45).

**Table 6.** Early sexual experiences by birth order in women and men aged 17–29 in Natsal-3 – multiple regression models adjusting for socio-demographic factors and family size.

	Women				Men			
Outcome variable	paOR* (95% CI)	P	faOR** (95% CI)	P	paOR* (95% CI)	P	faOR** (95% CI)	P
**Under 16 at first sex**								
First-born	1.00	0.1180	1.00	0.0676	1.00	0.003	1.00	0.0008
Middle-born	1.01 (0.80 – 1.28)		0.94 (0.73 – 1.22)		1.77 (1.32 – 2.37)		1.82 (1.31 – 2.52)	
Last-born	1.24 (1.00 – 1.55)		1.24 (0.99 – 1.54)		1.05 (0.81 – 1.35)		1.05 (0.81 – 1.35)	
**Partner relatively older at first sex**								
First-born	1.00	0.0965	1.00	0.3577	1.00	0.0354	1.00	0.0360
Middle-born	0.17 (1.05 – 2.63)		1.28 (0.78 – 2.08)		0.69 (0.39 – 1.23)		0.75 (0.38 – 1.45)	
Last-born	1.40 (0.89 – 2.21)		1.38 (0.88 – 2.18)		0.47 (0.27 – 0.84)		0.48 (0.27 – 0.84)	
**Either respondent or partner more willing at first sex**								
First-born	1.00	0.3673	1.00	0.3575	1.00	0.3468	1.00	0.3329
Middle-born	0.87 (0.64 – 1.19)		0.86 (0.61 – 1.20)		0.92 (0.55 – 1.53)		0.72 (0.42 – 1.23)	
Last-born	0.81 (0.60 – 1.09)		0.81 (0.60 – 1.09)		0.73 (0.46 – 1.15)		0.73 (0.46 – 1.14)	
**Did not use reliable contraception at first sex**								
First-born	1.00	0.1135	1.00	0.4048	1.00	0.2938	1.00	0.7321
Middle-born	1.22 (0.86 – 1.71)		1.04 (0.72 – 1.52)		1.35 (0.91 – 1.99)		1.18 (0.78 – 1.79)	
Last-born	0.86 (0.62 – 1.18)		0.84 (0.61 – 1.17)		1.04 (0.72 – 1.49)		1.03 (0.72 – 1.48)	
**Thinks should have waited longer for first sex**								
First-born	1.00	0.5870	1.00	0.6663	1.00	0.1300	1.00	0.3283
Middle-born	1.13 (0.89 – 1.42)		1.12 (0.87 – 1.44)		1.28 (0.91 – 1.78)		1.21 (0.83 – 1.76)	
Last-born	1.03 (0.82 – 1.29)		1.03 (0.82 – 1.29)		0.90 (0.65 – 1.25)		0.90 (0.64 – 1.24)	
**Non-autonomous reason for first sex**								
First-born	1.00	0.3978	1.00	0.3736	1.00	0.7730	1.00	0.8539
Middle-born	1.15 (0.85 – 1.57)		1.17 (0.84 – 1.65)		1.11 (0.74 – 1.66)		1.09 (0.70 – 1.69)	
Last-born	0.93 (0.70 – 1.24)		0.93 (0.70 – 1.24)		0.96 (0.66 – 1.41)		0.96 (0.66 – 1.40)	
**Not competent at first sex**								
First-born	1.00	0.4446	1.00	0.4816	1.00	0.0461	1.00	0.1287
Middle-born	1.03 (0.81 – 1.30)		1.01 (0.79 – 1.30)		1.30 (0.98 – 1.71		1.26 (0.93 – 1.71)	
Last-born	0.91 (0.72 – 1.11)		0.89 (0.72 – 1.11)		0.91 (0.71 – 1.17)		0.91 (0.71 – 1.17)	
**Pregnancy before age 18**								
First-born	1.00	0.0562	1.00	0.1729				
Middle-born	1.48 (1.04 – 2.09)		1.40 (0.96 – 2.01)					
Last-born	1.07 (0.76 – 1.51)		1.07 (0.75 – 1.51)					

* Partially-adjusted odds ratios: adjusted for age, education level, religion, ethnic group, quintile of adjusted index of multiple deprivation, parents’ social class, and family structure at age 14. ** Fully-adjusted odds ratios: adjusted for all socio-demographic factors and for number of siblings.

## Discussion

### Family involvement in sex education

Taken together, these data suggest that later-borns have reduced odds of reporting parental involvement in sex education, although patterns differed between men and women. Later-born men appear to find it more difficult than first-born men to talk to their parents about sexual matters during adolescence, whereas later-born women do not report such difficulties but nonetheless have reduced odds of having learnt about sex from their mother or father. Previous analysis using Natsal data has identified men as being less likely to report parental involvement in sex education (Macdowall et al. 2006); this analysis further identifies middle-born and last-born men as having reduced odds than first-born men of reporting parental involvement, suggesting that the difficulties parents face in discussing sex with their male children (Dilorio, Kelley, and Hockenberry-Eaton 1999; Wilson et al. 2010) may be exacerbated if male children are middle- or last-born. Given literature that suggests that closeness of the parent-child relationship (Wilson et al. 2010) and amount of time spent with children (Turnbull 2012) impacts upon parent-child communication about sex, it may be that previously-reported differences in parental investment by birth order – whereby parents expend less time and energy on their middle- and last-born children (Heer 1986; Kalliopuska 1984; Ware 1973) – may be implicated in the birth order disparities reported here.

All later-borns, both male and female, had higher odds of reporting learning about sex from siblings than first-borns, although the confidence intervals for the sibling analyses were wide. Whilst the positive impact of receiving school-based or parental sex education on sexual health outcomes has been noted (Macdowall et al. 2015), little research exists examining the specific effect of sex education from siblings on subsequent sexual health. However, sibling effects on sexual behaviour have been demonstrated: having sexually active siblings has been associated with permissive sexual attitudes, whilst sisters of pregnant and childbearing adolescents have also been described as being younger at initiation of sexual intercourse (Hogan and Kitagawa 1985). Whilst our analyses showed no differences in early sexual experiences by birth order in women, sibling effects on sexual health outcomes cannot be ruled out. It may be that the impact of sibling sex education is more nuanced than can be captured in the Natsal data, influencing emotional wellbeing and attitudes more than experiences or behaviours. Killoren and Roach (2014), for example, note the supportive role older sisters play in providing advice about dating and sexuality to their younger sisters; this may contribute to the lower odds of parental involvement in sex education in middle- and last-born women.

### The role of sibling number

The strength of association between birth order and a number of outcomes was reduced once sibling number was adjusted for; for example, no statistically significant association between being a middle-born child and pregnancy before 18 was shown after adjustment for number of siblings.Sibling number thus appears to act as a confounder of the relationship between birth order and some sexual health outcomes, as Steelman and Powell (1985) suggest. Previous research has identified sibling number as being of relevance for various outcomes relating to sexual health: Hogan and Kitagawa (1985) for example, found that sibling number was positively correlated with early sexual activity and increased odds of unintended pregnancy amongst Black adolescents in the USA.

The effect of sibling number has been well-documented, with Featherman and Hauser (1978) and Conley and Glauber (2005) both identifying negative effects of increased sibling number on educational attainment with each successive child. Several mechanisms may account for sibling number effects on psychosocial outcomes: first, large family size is associated with socio-economic disadvantage, which may not have been adequately adjusted for using the available socio-economic variables; secondly, interaction and communication between parents and children may be less intensive, since parental time has to be distributed more widely; and thirdly, parental supervision and discipline may be harder to achieve with a greater number of children. The latter two mechanisms relate to the dilution of family ‘resources’ such as parental time and attention, which has been widely cited as a principal cause of birth order differences in a range of outcomes, including educational attainment and delinquent behaviour (Cundiff 2013; Harkonen 2012). It could therefore be hypothesised that sibling number is an effect modifier of birth order, and that high sibling number compounds birth order-related dilution of parental interaction and supervision. Adjustment for sibling number is thus essential in any analysis of birth order effects, particularly given our results that demonstrate middle-borns report higher sibling numbers than first- or last-borns.

### Strengths and limitations

The data obtained from Natsal-3 were nationally representative in terms of gender, age and region, and closely match the 2011 census figures for England and Wales, whilst the use of probability sampling means that results obtained from Natsal-3 are likely to be generalisable to the general population (Erens et al. 2014). Even with sub-sampling of the overall Natsal-3 cohort, the sample used in this analysis was large and the birth order categories had similar numbers of participants. This increases the likelihood that there was sufficient power to detect differences between different birth order categories. To our knowledge, this is the first study of its kind to explore family involvement in sex education by birth order, and our analysis of early sexual experience provides a more complete picture than earlier literature focusing on single sexual risk behaviours.

There are some limitations of our analysis. Many odds ratios were reasonably close to the null value, suggesting that birth order effects may be small, even if statistically significant. Although adjustment was made for sibling number, the impact of birth order on the outcomes analysed may have been modified by a number of other sibling-related factors for which it was not possible to adjust. For example, although Kidwell (1981) and Heer (1986) suggest that research focusing on birth order as an explanatory variable must control for spacing of siblings, age of siblings was not specified in Natsal-3 and thus it was not possible to control for age differences between siblings. Gender of siblings, which was not investigated in this analysis, may also influence the effect of birth order: Haurin and Mott (1990) found a greater correlation in age at first sexual intercourse between same-sex than opposite-sex sibling pairs.

First-borns and last-borns in our sample had very similar socio-demographic characteristics, and reported similar sibling numbers. Caution must therefore be taken in interpreting the results for middle-born children as purely reflecting birth order. Whilst every effort was taken to adjust for socio-demographic characteristics and family size, the results for middle-born children may be due, at least in part, to unobservable socio-demographic differences.

## Conclusions

As an exploratory analysis, our work was intended to generate further research questions in a field that has had little attention thus far, and to draw links between birth order literature and studies of parental sex education. Our findings add context to previous work demonstrating gender disparities in family involvement in sex education (Tanton et al. 2015; Nolin and Peterson 1992) and identify later-born men as being particularly disadvantaged in comparison to male first-borns; future study of gender disparity in familial sex education should thus be mindful of the effect of birth order. Qualitative research would be useful to gain a broader understanding of the ways in which birth order effects manifest in parental involvement in sex education, since cross-sectional analysis alone cannot fully explore the processes underlying birth order effects. In the design of parental sex education programmes, it is important to consider how aspects of the parent-child relationship, including birth order effects, could be included in such programmes.

Whilst Tanton et al. (2015) have shown using Natsal data that siblings are a less important source of sex education than parents or school, our analysis demonstrates that siblings appear to be a significant source of sex education for middle- and last-borns. However, there is very little research exploring sibling sex education in comparison to the numerous studies exploring sex education received from parents (Crosby, Hanson, and Rager 2009; Walker 2001; Wilson et al. 2010). A qualitative approach would be well-suited to explore in more detail how learning about sex from siblings affects the development of attitudes and beliefs around sexuality, as well as how the experiences or advice of siblings might influence sexual behaviour. Older siblings appear to be an under-researched – and perhaps under-utilised – source of sex education for their younger siblings, and there is scope for greater consideration of their role in sex education and for increased inclusion of older siblings in sex education programmes.
